# Career opportunities in global health: A snapshot of the current employment landscape

**DOI:** 10.7189/jogh.05.010302

**Published:** 2015-06

**Authors:** Quentin Eichbaum, Adam Hoverman, William Cherniak, Jessica Evert, Elahe Nezami, Thomas Hall

**Affiliations:** 1 Vanderbilt University School of Medicine, Nashville, TN, USA; 2 Pacific Northwest University of Health Sciences, Department of Family Medicine, Yakima, WA, USA; 3 University of Toronto, Department of Family and Community Medicine, Toronto, Ontario, Canada and Bridge to Health Medical and Dental, Toronto, Ontario, Canada; 4 Child Family Health International and University of California at San Francisco, San Francisco, CA, USA; 5 Keck School of Medicine, University of Southern California at Los Angeles, CA, USA; 6 University of California at San Francisco, San Francisco, CA, USA

Recent decades have witnessed a burgeoning interest in improving health and health systems in low and middle–income countries (LMIC). With the increase in program funding came parallel increases in the number of university programs in the US and Europe offering concentrations or degrees in a global health field [1–6]. The changes have been brisk and substantial [5–8], and beg the question: What do we know about the global health job market? While a few studies have provided limited insights into the employment landscape, the last comprehensive attempt to answer this question was by Baker 30 years ago [9]. Key questions (as yet largely unanswered) about the current global health job market include:

How satisfactory is the numerical balance between job aspirants and job openings? Are the trend lines of aspirants and openings similar or divergent? Are we at risk of having too many job seekers or too few?

How good is the qualitative match between employer needs and training program outputs? Which competencies are in short supply [10–12]?

What are the contributions, and liabilities, of short–term trainee and volunteer participation in the workforce? How do their contributions fit into the larger picture of the global health workforce [5,10–12]?

Answering these questions will take substantial effort through carefully structured investigations to provide reliable answers. In this article, we present a limited pilot study through a targeted web–based job posting review that does not attempt to answer all these questions but sheds some light on the current landscape of employment opportunities in program management, clinical, and public health–related aspects of global health in international settings.

The investigative team consisted of five physicians, one with a doctorate in psychology, and another with a doctoral degree in Public Health. The team convened in March 2013. Review of online job postings occurred between November 2013 and January 2014. Websites with employment opportunities in global health were identified using the Google search engine. The terms, “Global Health Work,” “Global Health Jobs,” “Global Health Job Opportunities,” “Global Health Workforce,” “Global Health Hiring” were searched in August 2013.

These searches returned a large number of results with potential sources of job information. From this sizeable response, for feasibility and efficiency’s sake, an initial cohort of 14 websites were selected, limited to English language websites primarily affiliated with organizations in North America, and (if the site permitted access) to a regularly available and rotating list of job postings. A similar consideration in prioritizing this initial pilot list was the Google “PageRank” of each site. Page Rank is an objective measure of a citation’s importance that corresponds with users subjective idea of importance [13]. Over the course of the entire survey, 12 further sites were selected to accrue additional postings by applying the same inclusion criteria. The need for additional sites addressed cyclical pauses in available job postings on several sites. Global health workforce employment opportunities were described as positions that focus on health–related efforts in low– and middle–income countries (LMIC).

The investigative team developed a standardized selection and coding tool using a shared online document matrix. The tool allowed for easy categorization of a number of factors related to the job in question. 26 websites in total were selected for inclusion. Each investigator was assigned one high traffic website with frequent job postings and another with lower traffic and fewer postings. The six investigators then each reviewed a subset of the websites during two 6–week sampling periods. Each investigator retrieved a minimum of 10 job postings during each sampling period. The results were then tabulated and underwent basic statistical analysis.

**Figure 1:**
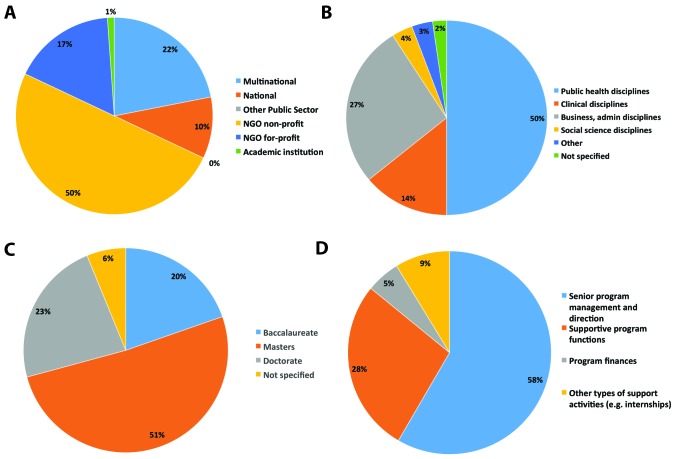
Depiction of survey results of career opportunities in global health. **A)** Breakdown of types of global health employers. **B)** The primary disciplines sought by employers. **C)** Highest academic achievement required or desired by employers. **D)** A sub–categorization of specific function desired in a program–related job.

In this limited, but wide–reaching review of online job postings that included 178 employment opportunities from 26 websites, key findings included:

67% (119/178) of the positions were in non–governmental organizations (NGOs) in both developed countries and LMICs. When combined with multinational organizations such as the World Health Organization (WHO) and the World Bank, the two employer types accounted for 89% (158/178) of the total (**Figure 1**, plate A).

14% (25/178) of the positions involved clinical disciplines primarily medicine. (**Figure 1**, plate B).

50% (89/178) of job posts included the request for applicants to have the kind of knowledge and skills normally acquired in schools of public health offering courses relevant to global health.(**Figure 1**, plate B)

51% (91/178) of the listed opportunities required at least a Master’s degree level of qualification or doctoral degree (23%, 41/178) (**Figure 1**, plate C).

**Figure:**
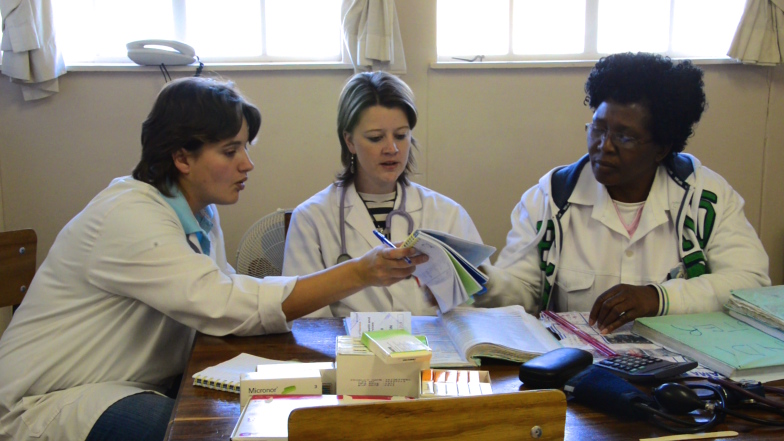
Photo: Courtesy of Trisha Pasricha, personal collection (from the documentary “A Doctor of My Own”, directed by T. Pasricha)

84% (149/178) of the positions were program–related. Program–related jobs included planning, program direction, finance, management and other supportive functions (not depicted but subcategorized in **Figure 1**, plate D).

**Figure 2:**
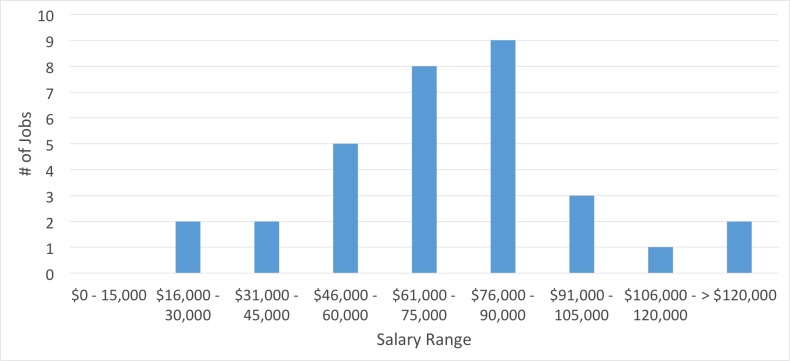
Distribution of global health jobs based on salary range.

The majority of program–related jobs were identified to be at the senior program management and direction level (58% (87/149) (**Figure 1**, plate D). Second most common were supportive program functions (28% 41/149) followed by other support activities (9% 13/149) and program financing (5% 8/149) (**Figure 1**, plate D). Salary information, which could provide a basis for assessing the strength of demand and for calculating a rate of return on a global health job, was provided in only 18% (32/178) of the job offerings. Of those listed, most (56%, 18/32) were in the US$ 61 000 – 90 000 range (**Figure 2**).

The size, characteristics and trends of the global health workforce and jobs available are largely unknown. Our pilot study of internet–based job postings provides a initial snapshot of one view of global health employment opportunities in international settings. Aside from highlighting the many as yet unanswered questions regarding the global health workforce, the study itself has limitations with respect to its specific focus on the job market. These include: small sample size, use of only English language job postings accessible on the internet, the scant salary and benefit information available, and the generally limited scope of positions in LMICs. The salary ranges available may be on the lower end as higher salary jobs may conceivably not be publicized. We did not attempt detailed analysis of the many discrete skills sought by employers, nor did we make follow up phone calls to employers to learn whether they readily filled the advertised positions and with the requisite qualifications.

Despite these limitations our findings have implications for the curricula of global health educational programs and to graduates seeking employment and career opportunities. For instance, our investigation draws attention to the importance of public health training and to program management skills. Global health programs should seek to include training in public health with an emphasis on leadership, planning, management, financial, communication, evaluation and related programmatic skills. Given that 74% of the jobs we surveyed required a Master’s degree or higher, the importance of advanced academic credentials is evident. This high level academic qualification has clear implications for students and trainees seeking a career in global health as they will be required to spend more time and tuition in academia before entering the job market. Given the ongoing increases in tuition costs for many undergraduate and advanced degrees, the average salaries offered may appear inadequate for those needing to repay student loans.

Our study suggests the importance of probing more deeply into the dynamics of the global health workforce, including how this workforce is trained and educated as well as the employment opportunities available following the completion of training. Pending an updated investigation along the lines of the Baker 1982 study, several interim studies might be considered: (1) studies to gain a better understanding of the content and the characteristics of global health (and related) training programs; (2) studies to understand the match between employer needs and applicant qualifications; (3) analyses of the likely trends and stability of the global health job market; (4) surveys of the Global South host countries to determine if training among visitors from the Global North adequately meets their needs; (5) analyses of the intersections between domestic and international employment opportunities, training and career paths. We welcome exchanging views with others interested in learning more about the global health workforce.
